# HDDM Hardware Evaluation for Robust Interference Mitigation †

**DOI:** 10.3390/s20226492

**Published:** 2020-11-13

**Authors:** Fabio Garzia, Johannes Rossouw van der Merwe, Alexander Rügamer, Santiago Urquijo, Wolfgang Felber

**Affiliations:** Satellite-Based Positioning Systems Department, Fraunhofer IIS, Nordostpark 84, 90411 Nuremberg, Germany; alexander.ruegamer@iis.fraunhofer.de (A.R.); santiago.urquijo@iis.fraunhofer.de (S.U.); wolfgang.felber@iis.fraunhofer.de (W.F.)

**Keywords:** global navigation satellite system (GNSS), interference, mitigation, high-rate DFT-based data manipulator (HDDM), discrete Fourier transform (DFT)

## Abstract

Interference can significantly degrade the performance of global navigation satellite system (GNSS) receivers. Therefore, mitigation methods are required to ensure reliable operations. However, as there are different types of interference, robust, multi-purpose mitigation algorithms are needed. This paper describes the most popular state-of-the-art interference mitigation techniques. The high-rate DFT-based data manipulator (HDDM) is proposed as a possible solution to overcome their limitations. This paper presents a hardware implementation of the HDDM algorithm. The hardware HDDM module is integrated in three different receivers equipped with analog radio-frequency (RF) front-ends supporting signals with different dynamic range. The resource utilization and power consumption is evaluated for the three cases. The algorithm is compared to a low-end mass-market receiver and a high-end professional receiver with basic and sophisticated interference mitigation capabilities, respectively. Different type of interference are used to compare the mitigation capabilities of the receivers under test. Results of the HDDM hardware implementation achieve the similar or improved performance to the state of the art. With more complex interferences, like frequency hopping or pulsed, the HDDM shows even better performance.

## 1. Introduction

Interference events in the global navigation satellite system (GNSS) frequency bands are increasing [1,2,3,4,5]. Hence, reliable interference mitigation methods are required to remove these undesired signals and ensure safe GNSS navigation. An ideal mitigation enhanced receiver should be robust against interferences and still be able to provide positioning, navigation, and timing (PNT) capabilities. Interferences have many different origins: they can be unintentional, such as spectrum sharing with distance measurement equipment (DME) [6], but also intentional, like in the case of privacy protection devices (PPDs) [7]. Consequently, many different waveforms cause interference [5], including signal modulations as pulsed noise, frequency-modulated continuous-wave (FMCW) (also referred to as “chirp” or “swept frequency” signals), frequency hopping, matched spectrum, or a combination of any of these. This diversity of interference waveforms make the development of a mitigation methods challenging—especially considering future waveform enhancements. Consequently, an interference mitigation algorithm should also be resilient to any new interference characteristics.

The high-rate DFT-based data manipulator (HDDM) algorithm has shown good potential for interference mitigation in software [8], and initial hardware evaluations [9]. Furthermore, it has also shown to be adaptable to other applications such as spectrum compression and overlay receivers [10]. The HDDM is similar to the frequency-domain adaptive filtering (FDAF) method, but with the fundamental difference that a fast Fourier transform (FFT) is calculated for every newly-received digital sample of the signal. Implementing an FFT each clock cycle is a challenging task, as most hardware-based FFTs reuse the same processing blocks through resource sharing. The calculation of the FFTs cycle-by-cycle requires a complete parallelization of the algorithm. This over-redundant calculation of the FFT allows this algorithm to achieve superior time selectivity making it more robust against pulsed signals without the loss of frequency selectivity. As such, this method is robust and effective against several different interference signals.

This paper presents the resource requirements and performance of a hardware implementation of the HDDM. Further, it extends the conference paper it is based on [9] by including evaluations with the HDDM on low size, weight, power, and cost (SWAP-C) and low bit-width receivers. The aim is to identify the scalability limits of the algorithm and application to low SWAP-C mass-market receivers in the future.

The hardware implementations are evaluated using different interference signals. This comparison benchmarks the algorithm to current industry capabilities. The HDDM algorithm presents a large versatility, since it shows good mitigation capabilities against FMCW, frequency hopping, and pulsed signals. In some cases, the mitigation is better than in the high-end market receiver. The efficient hardware implementation provides the possibility to include such a method in medium-end and high-end market receivers.

Section 2 presents some background to interference mitigation and describes several mitigation algorithms, including the HDDM. Section 3 describes the hardware implementation of the HDDM algorithm and some trade-offs to take into account. Section 4 outlines the experimental setup, with the results presented in Section 5, and discussed in Section 6. Finally, conclusions are drawn in Section 7.

## 2. Background

This section provides some background to the HDDM. Firstly, interference signal analysis methods are presented. Secondly, to better illustrate the differences between the HDDM and other similar algorithms, several known interference mitigation algorithms are presented. After that, the HDDM is discussed and compared.

### 2.1. Interference Signals Analysis

The spectral separation coefficient (SSC) is a straightforward theoretical impact assessment of interference signals [11,12]. It allows the analysis of interference in the absence of any mitigation methods. Advanced receiver-specific properties, such as considering the dynamic range of the analog-to-digital converter (ADC) or coherency between the pseudo-random noise (PRN) codes used by GNSSs, also exist [13]. However, these require specific information for the receiver and each signal. Therefore, they are considered outside the scope of this paper.

Theoretical interference mitigation performance analysis is not straightforward. Many mitigation methods are time-variant or non-linear which significantly increases analysis complexity. Furthermore, the mitigation algorithms behave differently against diverse interference signals. As an example, pulsed signals are simple to analyze: removing the entire signal when the pulse is present results in a carrier-to-noise density ratio ($C/N_{0}$) reduction proportional to the duty-cycle of the pulse [14,15]. Likewise, an analytical expression exists for the more complex filter-bank pulse blanking (FBPB) [16]. For another example, an infinite impulse response (IIR) notch filter (NF) can adequately remove interferences with static frequency responses, like single-tone continuous-wave (CW) signals [17,18,19,20]. In this case, altering the front-end response H(f) would accurately model the mitigation performance in the SSC method. However, as soon as the interference has some dynamics, for example, an FMCW (e.g., chirp signals), then the analysis becomes considerably more complicated.

Considering the aspects mentioned above, the SSC remains a fundamental analysis tool and provides a good baseline performance comparison for the non-mitigated case. Therefore, this method is discussed in more detail. The SSC $\kappa_{\mathrm{SSC}}$, and associated jamming resistance quality factor $Q_{j}$, describe the spectral similarity between an interference signal and a GNSS signal [11,12] and is defined as:

(1) $$\kappa_{\mathrm{SSC}}=\int_{-\infty }^{\infty }{\left|H(f)\right|}^{2}S_{s}\left(f\right)S_{i}\left(f\right)\;df\;,$$

where H(f) is the front-end transfer function of the receiver, $S_{s}\left(f\right)$ is the normalized power spectral density (PSD) of the GNSS signal, and $S_{i}\left(f\right)$ is the normalized PSD of the interference signal. The jamming resistance quality factor $Q_{j}$ is a normalization of the SSC. It is unit-less, and it is defined as:

(2) $$Q_{j}=\frac{\int_{-\infty }^{\infty }{\left|H(f)\right|}^{2}S_{s}\left(f\right)\;df}{\kappa_{\mathrm{SSC}}R_{c}}\;,$$

where $R_{c}$ is the chipping-rate of the GNSS signal. The lower $Q_{j}$ is, the more effective the interference. A matched spectrum interference is considered the most effective interference [12]: It has a theoretical $Q_{j}$ of 1.5 for a binary phase-shift keying (BPSK) signal, and a theoretical $Q_{j}$ of 2 to 4 for a binary offset carrier (BOC) signal. These values can be used as reference values when analyzing interferences. Finally, the effective $C/N_{0}$ (${\left(C/N_{0}\right)}_{\mathrm{eff}}$) relates to the interference-free $C/N_{0}$ and the jamming resistance quality factor $Q_{j}$:

(3) $${\left(C/N_{0}\right)}_{\mathrm{eff}}=\frac{1}{\frac{1}{C/N_{0}}+\frac{I/C}{R_{c}Q_{j}}}.$$

where I/C is the jamming-to-signal ratio (JSR). The JSR and interference-to-signal ratio (ISR) are considered to be the same metric in this study; therefore, the JSR will be use in the remainder of the paper.

The jamming resistance quality factor $Q_{j}$ for each interference for various GNSS signals in the GNSS L1 frequency band are determined in Section 4. It provides a baseline of the expected performance without interference mitigation for the results in Section 5.

### 2.2. Pulse Blanking (PB) Methods

Simple pulse blanking (PB) just replaces any large values by zeros [21,22,23]. It requires no multiplications and has the least computational complexity of any mitigation method [24]. It has good time but has no frequency selectivity.

**Figure 1:**
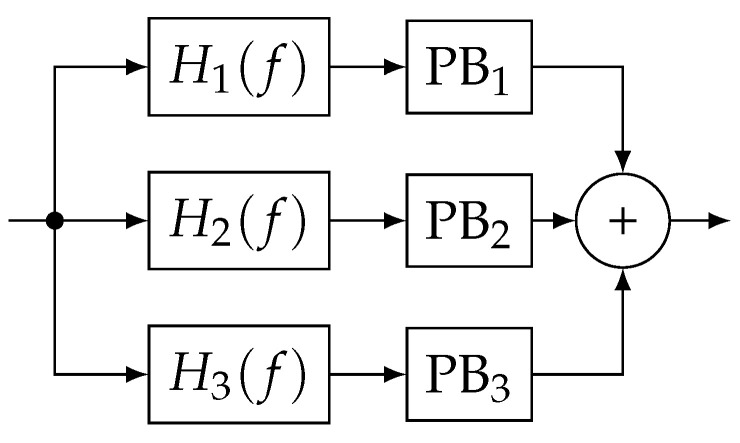
Block diagram for FBPB. ©IEEE. Reprinted, with permission, from [9].

Narrow-band (NB) PB uses a filter to spectrally limit a wide-band FMCW interference to be a quasi-pulsed signal [15]. PB then simply replaces the remaining large time values with zeros. FBPB is an extension of the NB PB [16]. FBPB uses a bank of filters, each followed by PB, to select only a part of the spectrum. Therefore, a wide-band (WB) receiver can mitigate multiple sub-bands simultaneously. Figure 1 shows a three-band example diagram for FBPB, consisting of $H_{n}\left(f\right)$ individual filters and ${\mathrm{PB}}_{n}$ pulse blankers.

Another approach is to use non-linear functions [25,26,27] instead of PB. The benefit of this approach is that some information is conserved. However, non-linear functions increase significantly the complexity, making them often unsuitable for hardware implementations.

The benefit of the FBPB is the time response available due to PB: as no down-sampling exists, no temporal loss is perceived. The limitations of the FBPB relate to the filter design. Better performing filters require more filter-coefficients and larger word-lengths which increase computational complexity.

### 2.3. Notch Filter (NF)

If the interference signal has a single spectral peak (i.e., a tone or CW signal), then an NF can be used to remove it. The NF is usually implemented with an IIR filter [17,28], such that high interference suppression can be achieved while removing as little as possible from the rest of the unaffected spectrum. CW signals are commonly caused through harmonics and inter-modulation products of the radio-frequency front-end (RFFE) oscillators—often called spurious tones. By using multiple cascaded notch filters, multi-tone signals can also be removed.

An effective mitigation method against chirp like signals is adaptive notch filtering (ANF) [17,18,19,20,28,29,30]. The notch frequency is adapted, such that it can follow the frequency modulation of the interference. The two popular adaption methods are the least mean squares (LMS) approach [19], and the frequency-locked loop (FLL) approach [30]. Under specific parameter selections, these algorithms are the same [29]. Some limitations with ANF include the adaption time, frequency discontinuities when the interference “jumps” to new frequencies, switching of the filter on or off, and the ineffectiveness against other types of interferences. One method to overcome some of these limitations is the wavelet-based adaptive notch filter (WANF) [31].

The IIR requires little processing resources, making an NF straight forward to implement. However, the non-linear response may cause instabilities. Further, the restricted adaptability to different interference types limit this approach to CW and simple FMCW signals. An ANF requires several processing operations for each new data sample, which cannot simply be parallelized because of the feedback stage. Consequently, it has high timing requirements, making it often unsuitable for WB receivers.

### 2.4. Frequency Domain Adaptive Filtering (FDAF)

**Figure 2:**
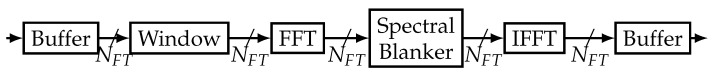
Block diagram for FDAF. ©IEEE. Reprinted, with permission, from [8].

The FDAF method transforms the signal to the frequency domain via a discrete Fourier transform (DFT) (often an FFT for processing efficiency), and removes some of the spectral components before transforming the signal back to the time domain with an inverse discrete Fourier transform (IDFT) (also with an inverse fast Fourier transform (IFFT)) [14,32,33]. As this method processes a block of data at a time, it is a batch-wise method. Figure 2 shows the block diagram of the FDAF. The DFT size $N_{FT}$ indicates the batch size of the algorithm. The spectral peak can be removed through the use of a power detector or by selecting the most significant number of peaks. Setting the spectral value to zero removes the information.

One downside of FDAF is the limited control of side-lobes. Usually, windowing before the FFT suppresses the side-lobes, but it causes unwanted temporal deformations after reconstruction. A method around this limitation is to use a channelized architecture, such as a poly-phase filter-bank [14,25]; however, it significantly increases computational complexity through the added filter.

**Figure 3:**
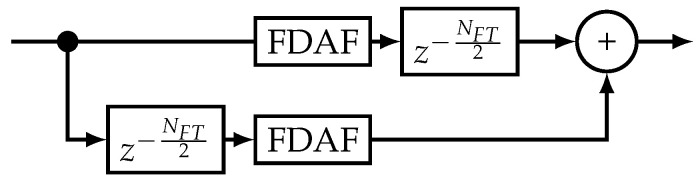
Block diagram for Dual-FDAF. ©IEEE. Reprinted, with permission, from [8].

A performance improvement of the FDAF is to use two parallel FDAF processing blocks with an offset [24,34]. This method is the dual-channel frequency-domain adaptive filtering (Dual-FDAF). Figure 3 shows the block-diagram for Dual-FDAF. The usage of two processing blocks smooths out any discontinuities, suppresses ringing effects, and allows the efficient use of windowing methods. Furthermore, as the processes overlap in time, this method is also more effective against shorter pulsed signals than the standard FDAF. The drawback of this method is that it requires approximately twice the processing and memory resources. In addition, in the ideal case, the windowing method should have the property of constant amplitude with overlapping windows, to limit temporal deformations.

### 2.5. Advanced Mitigation Methods

Many mitigation methods exist in the literature [14,27,35,36,37,38,39,40,41,42]. These include filter banks, Fourier methods, wavelet transforms, the Karhunen-Loève transform (KLT), and interference modeling methods. Although many methods have excellent results, they are often limited by the adaptability to hardware implementations due to non-linear operations, recursive processes, excessive memory and buffering requirements, high processing word-lengths including floating point operations, high computational complexity, or they are only applicable to certain interference types [24]. Complicated architectures which consists of several simultaneous mitigation algorithms, which work in parallel and only selects the best output also exist [43]. However, these present even more challenges for hardware adaptions.

These methods are commonly implemented using software-defined radio (SDR) platforms. These platforms consist typically of a hardware (HW) RFFE, which transfers samples to a personal computer (PC), where the interference mitigation and the GNSS are performed in post-processing. In such implementations, the front-end provides one order of magnitude less bandwidth and performance than embedded HW receivers. For example, in [44], the sampling frequency is 4 MHz. This is not suitable to process large bandwidth GNSS signals like high-order BOC or alternative binary offset carrier (AltBOC), or to receive on the same digital sample stream signals with a few MHz intermediate-frequency (IF) difference. Scaling of algorithms to higher sample-rates are not always straightforward. In some cases, only the sample-rate needs to be increased, causing a linear increase in processing complexity. However, if certain properties need to be maintained, e.g., keeping the same frequency resolution in Fourier based mitigation methods, then the algorithms typically scale non-linearly. As an example, a 256-point FFT scaled up by a factor 8 to keep the same frequency resolution but a factor 8 higher sample rate, requires 11 times more resources. This is 1.375 times more operations.

The software (SW) can be run on the desktop machine in post-processing, negating real-time or portable requirements. Such SW is not power efficient nor memory optimized, and cannot be easily ported to embedded HW receivers with limited memory, size and power consumption requirements (low SWAP-C systems). Therefore, there is often a significant gap in the performance and requirements between the theoretical state-of-the-art SW concepts and HW solutions.

Finally, the new emerging trend in interference mitigation is the use of machine learning (ML) methods [45,46]. These methods promise good performance against interferences. However, they have high processing demands when training the ML models, require significant training data to generalize, and can only successfully mitigate interferences which where represented inside the training data. Lastly, this technology is still emerging and the best approaches, architectures, and practices have not yet been identified. Therefore, it is not yet sufficiently mature and currently limits hardware implementation and optimization.

### 2.6. High-Rate DFT-Based Data Manipulator (HDDM)

**Figure 4:**
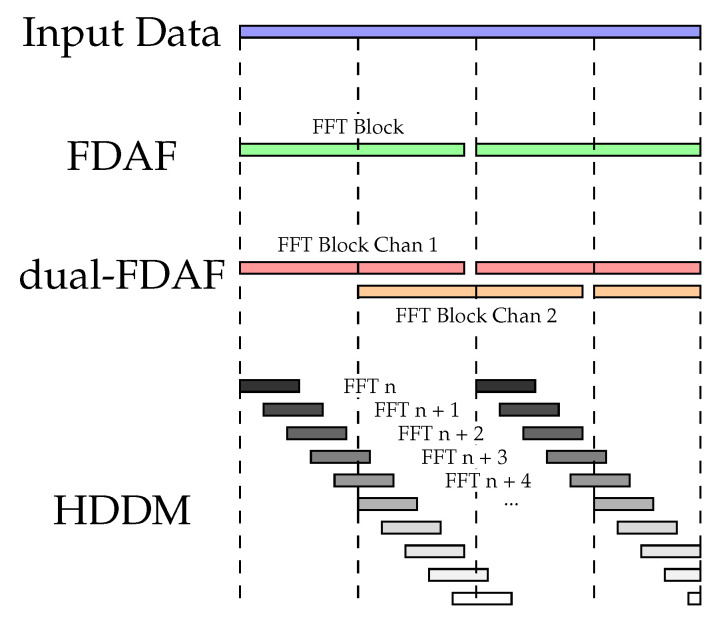
FFT block selection comparison between different algorithms. ©IEEE. Reprinted, with permission, from [9].

The HDDM implemented in this work builds on previous publications [8,9,47]. It uses an FFT to transform time-domain data into the frequency domain. These transformed data are then manipulated (e.g., for interference mitigation, PB is applied), and transformed back to the time domain using an IFFT. It is similar to the FDAF. However, HDDM calculates an FFT for every new input data sample, and not block-wise like FDAF, or interleaved block-wise like the Dual-FDAF. Figure 4 demonstrates the block selection and differences between the algorithms.

**Figure 5:**
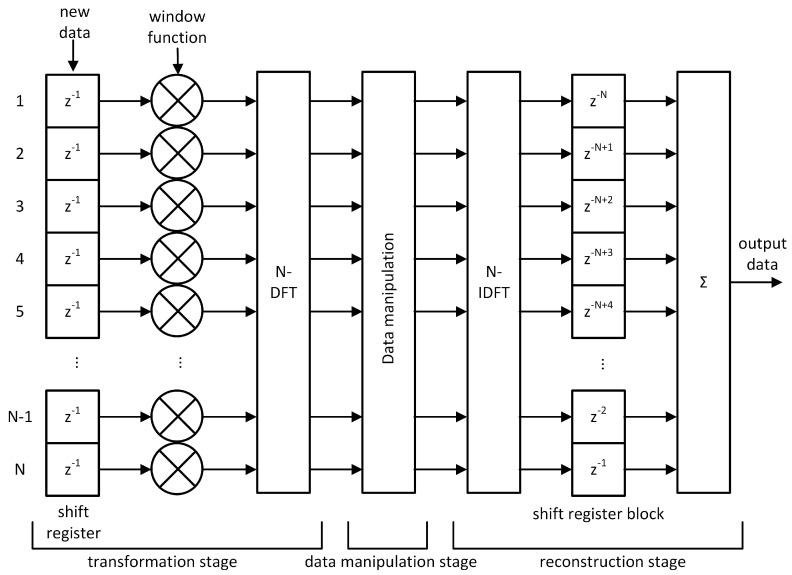
HDDM block diagram. ©IEEE. Reprinted, with permission, from [9].

The HDDM requires a shift-register before the FFT and a combiner after the IFFT. Using multiple delayed FFTs is not a new concept, as this also forms the basis for the Welsh transform for spectral estimation [48], as well as the basis for the short-time Fourier transform (STFT) [49]. The recombination to reconstruct the signal is the new development of the HDDM. A triangular delay buffer aligns all streams after the IFFT stage. This is composed by a sequence of registers, which delay each IFFT output by a different amount of clock cycles. The first output is delayed by the maximum amount of clock cycles, which corresponds to the size of the IFFT, i.e., 32 clock cycles. The last output is delayed by one clock cycles. The delayed outputs are then added together to generate one sample per clock cycle. Figure 5 shows the block diagram for the HDDM.

As the FFT is calculated for each new sample, it significantly increases the processing requirements for this method. Therefore, smaller FFT sizes are selected in comparison to the FDAF for the same resources. This oversampling facilitates improved mitigation capabilities and reconstruction of the signal. Furthermore, the use of the triangular register and combiner smooths out any signal distortions caused by discontinuities during its processing.

The oversampling of HDDM is more time-selective than achievable with FDAF, as it has a similar temporal response as the FBPB. Therefore, proving superiority in comparison to the FDAF. The FFT requires no filter design in contrast to the FBPB. Therefore, this method requires less effort in development and implementation. However, this implies that the FFT size limits the frequency selectivity. The frequency response of the system can still be altered by selecting appropriate windowing functions. The window function determines how frequency channels overlap and how much the side lobes are suppressed. The triangular register and combiner implicitly weigh the contributions of each reconstruction channel. Hence, the temporal effects of the window function are removed at the end of processing. What happens here is similar to the dual-FDAF. However, in the HDDM, any windowing function can potentially be used.

The FFT results in equally spaced frequency bins with the same bandwidth. Consequently, a flat-spectrum signal has the same power in every bin, and the same threshold for PB can be selected for all bins. This property makes threshold setting simpler in comparison with FBPB, as its time-domain filters may have different pass-band widths and filter gains, requiring different detector thresholds. This further demonstrates the simplicity of the HDDM in terms of design specification, implementation, and run-time control.

An NF has excellent frequency selectivity when compared to the HDDM. Therefore, an NF will outperform the HDDM against CW signals. However, combining a simple NF with the HDDM results in an ideal synergy of the two methods. It is recommended to precede the HDDM with at least one NF to remove any single-tone CW signals.

**Figure 6:**
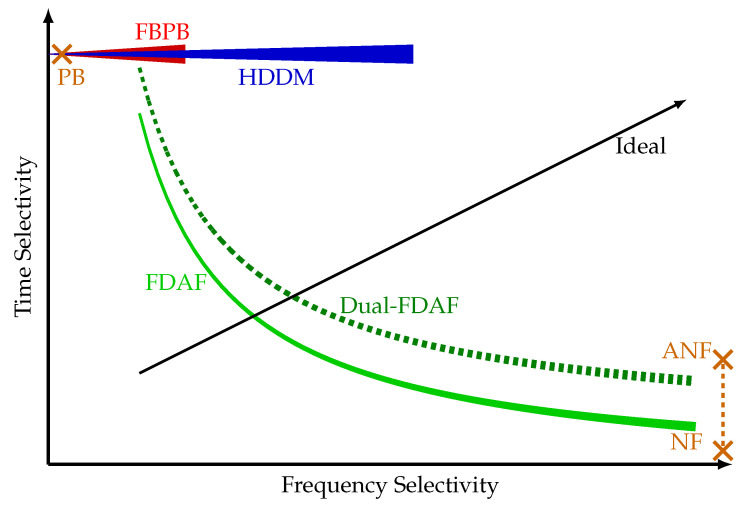
Conceptual comparison of mitigation algorithms, with the line thickness representing the computational complexity of different algorithms. Single points are relatively simple algorithms where the complexity is not indicated. This symbolic plot serves to provide an intuitive understanding of the different algorithms.

Figure 6 shows a conceptual ( The values in this plot represent the authors’ subjective understanding and interpretation of the algorithms.) comparison of the frequency and time selectivity of the algorithms. This figure aims to provide an intuitive comparison of the trade-off between the various algorithms. The exact performance of each algorithm varies based on the platform, implementation, and design goals. The line thickness also symbolizes the processing complexity of the algorithms. PB, NF, and ANF are only represented by single points, as these algorithms have limited scalability. The two extremes are PB, with high time selectivity, and NF with high frequency selectivity. FDAF presents a trade-off between these two, depending on the FFT size selected. The Dual-FDAF improves the FDAF at the cost of complexity. Both the FBPB and HDDM start with good time selectivity and improve in frequency selectivity as the implementation complexity increases. At a certain point, FBPB is limited by implementation complexity, but the HDDM is still feasible. Lastly, ANF has limited improved time selectivity compared to a simple NF, depending on the interference type. The ideal interference mitigation algorithm should provide both time and frequency selectivity. However, this is limited by the time-frequency trade-off and the dynamics of the interference signal. This plot emphasizes that each algorithm exhibits benefits compared to others and that no single mitigation algorithm is absolutely superior. A multi-algorithm approach can address the shortcomings of the individual algorithms.

In summary, the HDDM is an over-sampled version of the FDAF to achieve the time selectivity of the FBPB, simultaneously improving the frequency selectivity and the ease of design. Hence, this method is robust and straightforward to implement, but still flexible. The only tuning and calibration requirements are the selection of the windowing function and the threshold for PB.

## 3. Implementation

**Figure 7:**
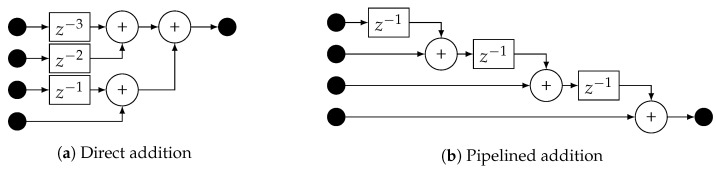
Comparison of triangular register methods. ©IEEE. Reprinted, with permission, from [9].

The HDDM hardware module has been designed in register-transfer level (RTL) code (i.e., very-high-speed integrated circuit hardware description language (VHDL)), allowing the implementation on different field-programmable gate array (FPGA) technologies or even as an application-specific integrated circuit (ASIC) intellectual property (IP). Figure 5 depicts the architecture. The central processing blocks are the DFT and IDFT, both based on a standard Cooley-Tukey radix-2 FFT core [50]. The FFT core size, bit-width, and type (direct or inverse) can be configured using VHDL generics. The maximum supported size is currently 32 points (i.e., HDDM-32), which means that the spectrum of the incoming signal is divided into a maximum of 32 bins of $f_{s}$/32 Hz each, where $f_{s}$ is the sampling frequency. Each frequency bin does independent PB mitigation. A control signal determines PB thresholds. In a system-on-chip (SoC) architecture, this signal can be driven using a memory-mapped register, which also allows the possibility to control the module via software. In addition to the FFT, the windowing and the reconstruction modules also require appropriate care. The window function is an approximated Hamming window. However, a combination of bit-shifts and additions constructed the window function, as opposed to generic multiplications. This way, resource-expensive multipliers are replaced by simple bit manipulations, avoiding sparse digital signal processor (DSP) blocks in FPGA implementations, too. Regarding the triangular reconstruction, the structure has been transposed from a direct addition to allow pipelining. Figure 7 compares the direct addition to pipelining.

The HDDM hardware module is implemented on three different FPGA-based GNSS dual-band receivers to evaluate resource utilization and power consumption for each specific implementation. All three receivers are based on Xilinx SoC devices. An Acorn RISC Machine (ARM) processor performs the receiver management in software and closes the GNSS tracking feedback loops. Dedicated hardware modules are implemented in the programming logic (PL) taking care of the signal conditioning, interference mitigation, and correlations between the signal and the replica PRN codes.

**Figure 8:**
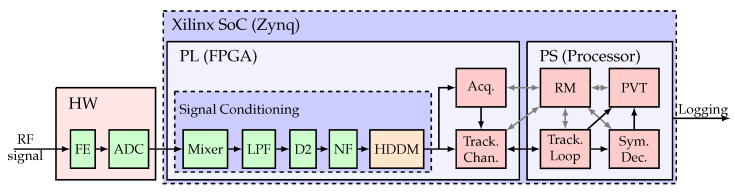
Hardware flow diagram.

The general receiver architecture is depicted in Figure 8. Each receiver consists of three parts. First, the RFFE consists of hardware (HW) components, does the analog radio-frequency (RF) processing, and digitization with an ADC. Second, the PL is implemented on the FPGA part of a Xilinx SoC. The signal conditioning is included on the FPGA, and it consists of a mixer, a low pass filter (LPF), decimate by factor 2 (D2), an NF and the HDDM. An acquisition engine (Acq.) and the tracking channels (Track. Chan.) are also implemented on the FPGA to allow efficient real-time processing. Third, the processing system (PS) does the high level GNSS processing, including the receiver manager (RM), the tracking loops (Track. Loop), symbol decoding (Sym. Dec.), and the position, velocity, and time (PVT) calculation.

The Receiver #1 is characterized by a discrete RFFE, a 14 bit ADC and a Xilinx Zynq7045 SoC device. The ADC delivers 14 bit real samples at a frequency of 216 MHz. These samples are down-converted using a $f_{s}$/4 mixer, filtered by a LPF and down sampled by factor two. At this point the signal is characterized by a sampling frequency of 108 MHz. The signal is then processed first by a notch filter and then by the HDDM for interference mitigation. The resulting signal is sent to GNSS acquisition and tracking modules. At each processing stage the amount of available quantization (i.e., bit) is optimized to limit the resource utilization but at the same time to preserve the dynamic range.

**Figure 9:**
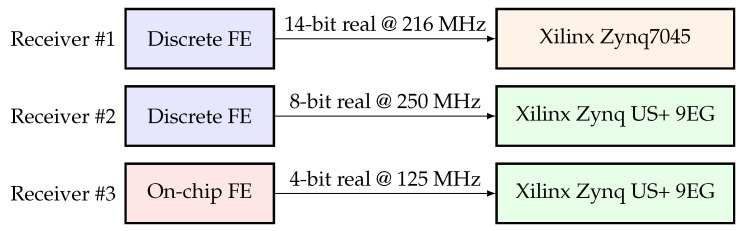
Receiver comparison.

The Receiver #2 is characterized by a discrete miniaturized RFFE, an 8 bit ADC and a Xilinx ZynqUS+ 9EG SoC. The ADC delivers an 8 bit real signal at a frequency of 250 MHz. The signal is then processed by a similar signal-conditioning chain. Differently from Receiver #1, Receiver #2 equips a coordinate rotation digital computer (CORDIC) mixer (due to the different analog RFFE local oscillator (LO) frequency) and a down-sampling factor four. The base-band signal is characterized by a sampling frequency of 62.5 MHz. The Receiver #3 uses the same Xilinx ZynqUS+ 9EG SoC as receiver #2. However, it equips an on-chip RFFE with an integrated 4 bit ADC [51]. The signal conditioning is similar to the one of receiver #2, but the input signal is here sampled at 125 MHz and down-sampled by factor two. The base-band signal is also characterized by a sampling frequency of 62.5 MHz. Figure 9 shows the difference between the receivers.

For all the three receivers, the internal word length of the PB stage is 12 bit. In the presence of an interference the incoming signal is characterized by an increased dynamic range, therefore the pre-mitigation stages have to provide a higher amount of bits than the one normally used by a signal without interference. However, after removing the interference with the PB stage, the bit-width can be safely decreased, without the risk of clipping or distortion. Therefore, for the IFFT and reconstruction stage, the word length is reduced to 8 bit in the first receiver and 4 bit in the other two. Additionally, a basic NF is implemented before the HDDM. The notch frequency is determined by taking periodic snapshots of data, calculating the Fourier transform of the snapshots, and searching a peak, therefore the notch setting is relatively slow and is only suitable for static single-tone interferences. The configurations are summarized in Table 1.

The signal conditioning resource utilization of the three implementations is provided in Table 1. The resources are divided to the various components for signal conditioning and shows both the look-up table (LUT) and DSP resource usages. The table includes the power estimation of the PL.

The logic in Receiver #1 is characterized by the highest resource utilization, because of the higher amount of bits used in the signal conditioning chain. The HDDM in Receiver #2 uses about the same number of DSP blocks in Receiver #1, but a slightly reduced amount of LUTs. The HDDM in Receiver #3 on the contrary, uses less DSP blocks than #2, but the same amount of LUTs. This trend is justified by the different dynamic range supported in the three receivers, but the usage of different resource types is related to the optimizations performed by the PL synthesis tools. This can be explained by assuming that multiplications with lower bit-widths are more efficiently implemented using LUTs than DSP blocks. The same can also be observed in the LPF resources. The NF, on the contrary, employs similar LUTs and DSP blocks in the three cases. The reduced resources for the mixer in Receiver #1 is justified by the use of a fs/4 instead of a CORDIC one. Regarding the power consumption, the dynamic power consumption shows a similar trend as the resource utilization. The power consumption estimation is obtained using the FPGA vendor tools and refers to the entire PL. This includes also the dedicated GNSS modules. However, since we use the same modules with the same parameters across the three systems, the difference in the comparison is mainly related to the bit-widths and the sampling frequency used. The power consumption amounts to 4.3 W for the Receiver #1, 2.5 W for the #2 and 2.0 W for the #3. This estimate does not take into account the processor part and the SW used to close the tracking loops and calculate the PVT.

As a comparison, the mass-market commercial-off-the-shelf (COTS) receiver is entirely powered over USB2.0 (i.e., less than 4.5 W) as well as the the high-end one. This shows that the HDDM receivers are comparable with industry standards in terms of power usage.

## 4. Experimental Setup

**Figure 10:**
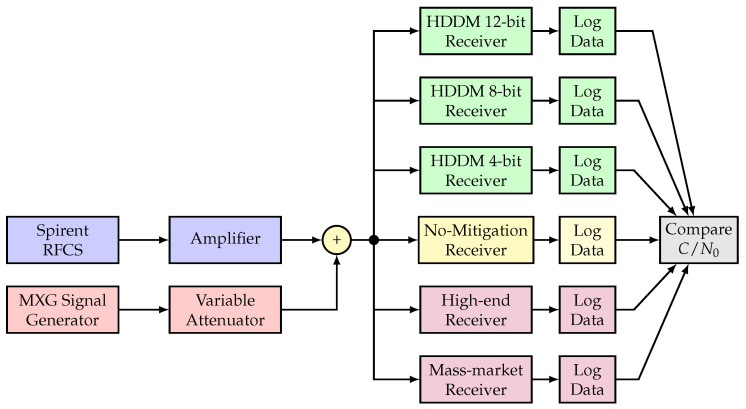
Experimental setup. ©IEEE. Adapted, with permission, from [9].

Figure 10 shows the experimental setup. A Spirent radio-frequency constellation simulator (RFCS) generates GNSS signals for a controlled laboratory setup, and an amplifier increases the signal power to compensate for subsequent losses (e.g., cables, splitters, etc.). Interference signals are generated in the laboratory and passed through a variable attenuator to evaluated different JSRs. The GNSS signals are combined with the interferences. The combined signal is passed to the three HDDM enabled receivers, a receiver with no interference mitigation, a mass-market COTS receiver, and a COTS high-end receiver. The two COTS receivers are described in Table 2.

The ”no mitigation receiver” uses the same hardware as the 14 bit HDDM, but with the HDDM bypassed. The estimated $C/N_{0}$ from each receiver assesses the receivers’ robustness.

**Figure 11:**
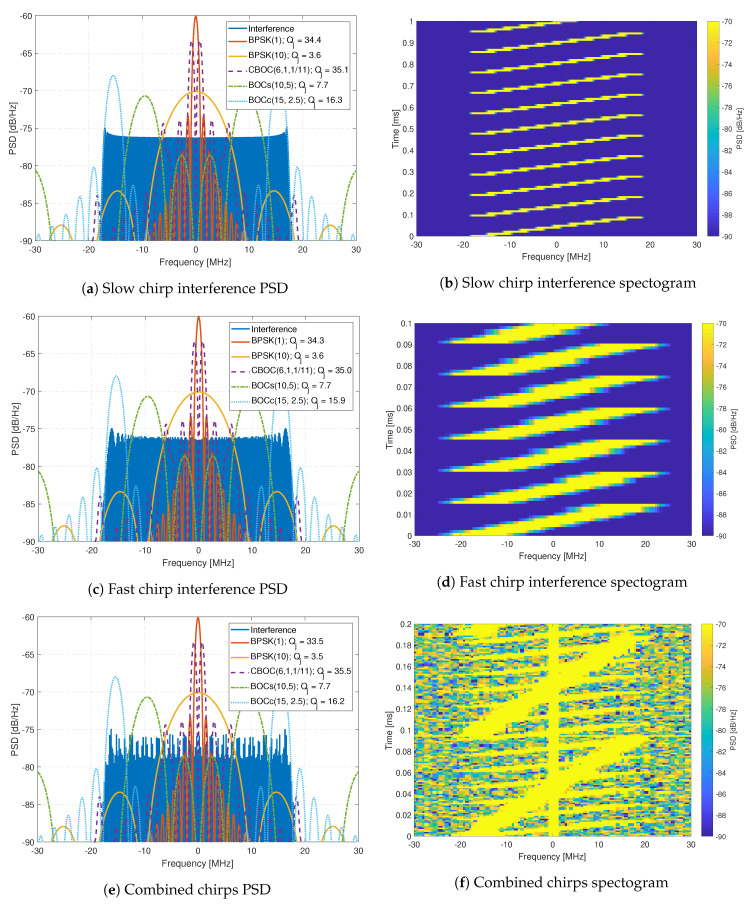
Analysis of chirp interferences.

**Figure 12:**
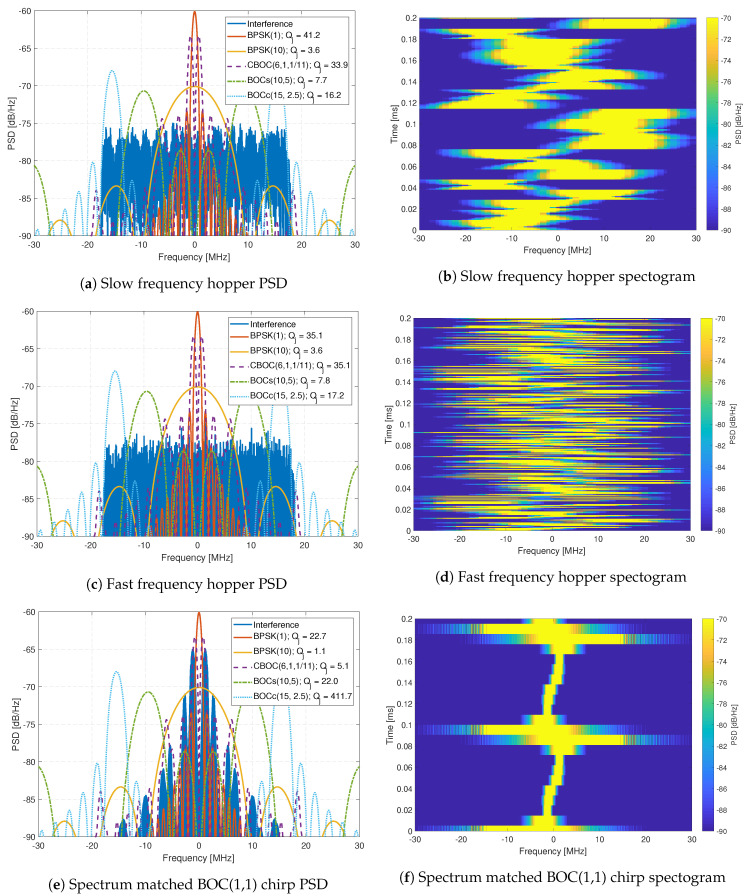
Analysis of complex FM interferences.

**Figure 13:**
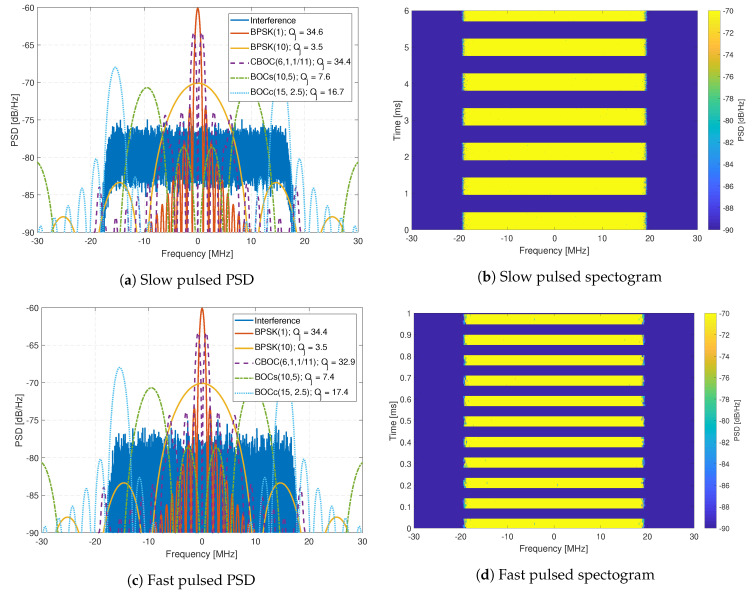
Analysis of pulsed FM interferences.

The interference signals are composed of eleven generated interference signals, which are replayed using an Agilent MXG-series Vector Signal Generator at a level of -10 dBm. The generated interference signals include wide-band chirp signals (Figure 11), complex frequency-modulation (FM) signals like frequency hopping signals (Figure 12), pulsed signals (Figure 13), and single-tone CW signals. These interference signals are chosen as they represent different scenarios, stressing the resilience capabilities of all receivers. Figure 11, Figure 12 and Figure 13 show the PSD compared to common GNSS signals and the spectrogram (also referred to as a waterfall diagram) for the interference. The jamming restive quality factor $Q_{j}$ between each GNSS signal and interference are also shown in the legend of the figures. It allows to determine the impact of non-mitigated interference. Here is a list of interference with a summary of their properties:

- Interference #1: Slow moving wide-band linear chirp with 35 MHz bandwidth and a chirp repetition rate of 100 μs (Figure 11a,b).
- Interference #2: Fast moving wide-band linear chirp with 35 MHz bandwidth and a chirp repetition rate of 16 μs (Figure 11c,d).
- Interference #3: Combined #1 and #2 interference signals (Figure 11e,f).
- Interference #4: Medium moving out-of-band linear chirp with 10 MHz bandwidth and a chirp repetition rate of 100 μs, this chirp does not overlap with the GNSS signals being tested; however, it still limits the receivers dynamic range.
- Interference #5: Slow frequency hopper with a dwell time of 10 μs and a frequency range of 35 MHz (Figure 12a,b).
- Interference #6: Fast frequency hopper with a dwell time of 1 μs and a frequency range of 35 MHz (Figure 12c,d).
- Interference #7: Slow matched spectrum chirp for a BOC(1,1) signal, with 35 MHz bandwidth and a chirp repetition rate of 100 μs (Figure 12e,f).
- Interference #8: Slow pulsed noise signal, with 35 MHz bandwidth, 500 μs pulse width, and 50% duty cycle (Figure 13a,b).
- Interference #9: Fast pulsed noise signal, with 35 MHz bandwidth, 1 ms pulse width, and 50% duty cycle (Figure 13c,d).
- Interference #10: Single-tone CW at 1 575.42 MHz, i.e., in the center of the GPS L1 frequency band.
- Interference #11: Single-tone CW at 1 576.42 MHz, i.e., 1 MHz above the the center of the GPS L1 frequency band.

The variable attenuator starts each test at 70 dB attenuation. At this level, the interference source is significantly lower than the noise. Hence, the interference-free performance of each receiver can be taken as a reference. This power level also allows the receivers to achieve stable tracking before the interference has a significant impact. The attenuation is decreased by 1 dB steps every 10 s until it reaches an attenuation of 0 dB. Then the attenuation is increased again at 1 dB steps per 10 s intervals, back to 70 dB. Therefore, each test is 23 min, with the maximum interference power after 11.5 min. The slow change of interference power allows the stable estimation of the performance of the receivers. The down-ramp phase (i.e., the first half of each test) evaluates the tracking sensitivity of each receiver. Both interference mitigation methods and high-performance tracking capabilities determine at which point the receiver loses lock. The up-ramp phase (i.e., the second half of each test) determines the acquisition sensitivity when the receiver is capable of regaining the GNSS signal for processing.

A spectrum-analyzer determined the interference to noise density ratio ($I/N_{0}$) to be 115 dBHz with a CW signal set at the maximum interference transmission power. The maximum $I/N_{0}$, the attenuation A(t), and the $C/N_{0}$ at the start of each test determine the JSR:

(4) $${\left(I/C\right)}_{\mathrm{estimated}}\left(t\right)\left[\mathrm{dB}\right]={\left(I/N_{0}\right)}_{\max}\left[\mathrm{dBHz}\right]-{\left(C/N_{0}\right)}_{\mathrm{start}}\left[\mathrm{dBHz}\right]-A\left(t\right)\left[\mathrm{dB}\right]$$

The estimated JSR is shown in each plot as a reference.

The HDDM receivers are compared to a COTS mass-market and a COTS high-end receiver. The latter is is known for its high-performance mitigation capabilities. This comparison facilitates the evaluation against the current low SWAP-C and high-performance state of the art. The tracking performance for a single satellite from Galileo with the E1BC signal is analyzed in the results. The selected satellite has a nominal $C/N_{0}$ between 48 and 50 dBHz. All receivers employ joint data (E1B) and pilot (E1C) tracking. The $C/N_{0}$ values, as reported by each receiver at a rate of 1 Hz, are used to assess the performance of the receivers. An averaging filter of ten values (i.e., 10 s integration) smooths the reported $C/N_{0}$ for clearer plotting and interpretation of the graphs.

## 5. Results

**Figure 14:**
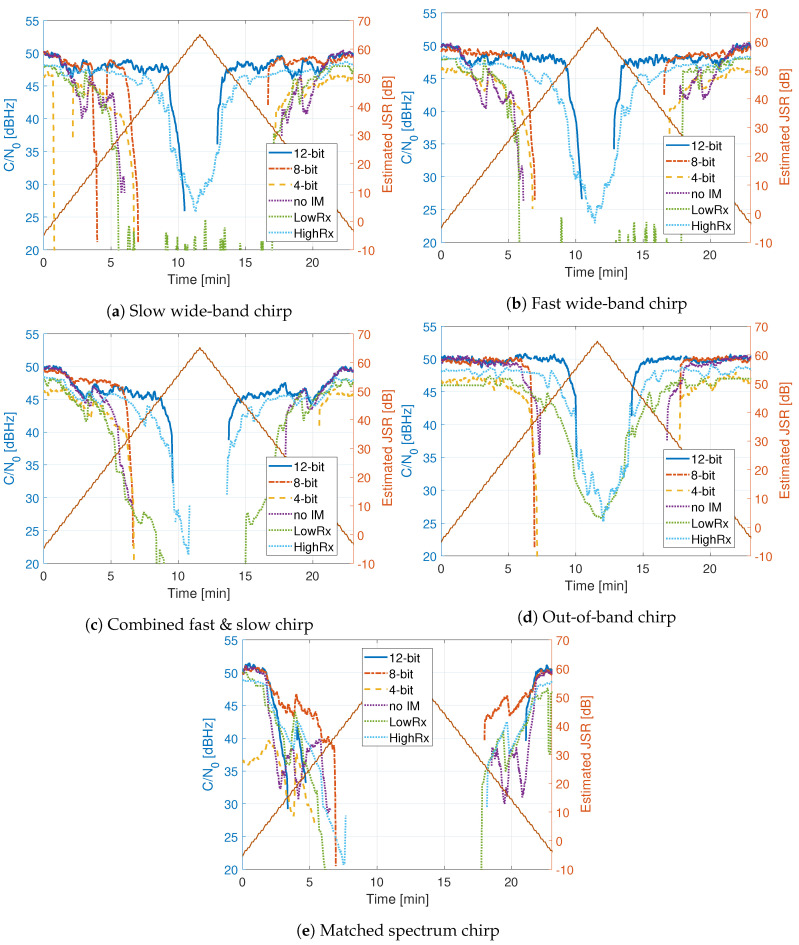
Comparison of tracking outputs for chirp signals.

**Figure 15:**
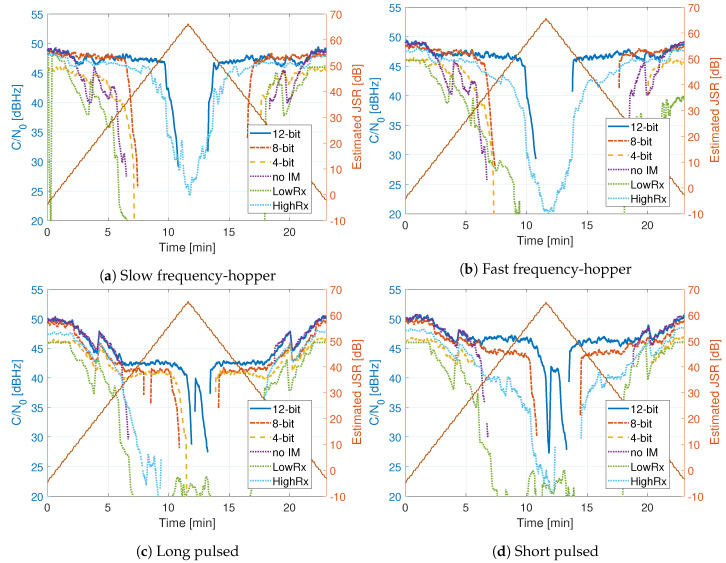
Comparison of tracking outputs.

**Figure 16:**
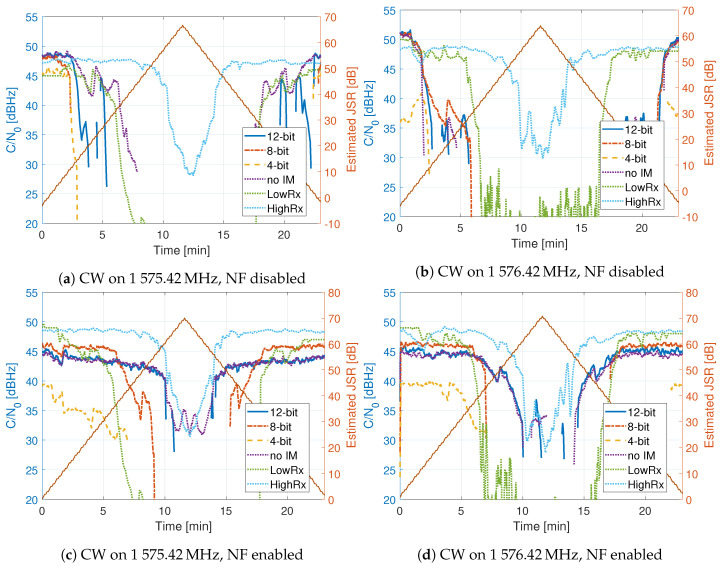
Comparison of tracking outputs.

Figure 14, Figure 15 and Figure 16 show the comparison of the receiver outputs to each interference. The HDDM receivers are indicated with “12 bit”, “8 bit”, “4 bit” in the legend. The no mitigation receiver is denoted as “no IM”, the mass-market receiver as “LowRx”, and the high-end receiver as “HighRx”. When there is no interference, or when the JSR is low, all receivers have comparable performance, with differences below 5 dB. The 12 bit HDDM has improved performance with respect to the 8 bit and 4 bit implementations, showing the significance of the word lengths for the algorithm. Furthermore, in most situations, the 12 bit HDDM has the best performance, followed by the high-end receiver, then the 8 bit HDDM. The no-mitigation receiver does slightly better than the mass-market receiver. It shows that the mass-market receiver has probably no or only basic interference mitigation capabilities.

Figure 14 shows the $C/N_{0}$ for the chirp signals. For the two chirp signals (Figure 14a,b), the high-end receiver and the 12 bit HDDM have similar performance. However, the high-end receiver stays better in lock at high JSRs. The combined chirp (Figure 14c) reduces the performance of all receivers. However, similar trends to single chirps can be seen. The out-of-band chirp (Figure 14d) still affects all of the receivers, even though it does not overlap in the same bandwidth as the GNSS signals. It shows the dynamic range sensitivity of the receiver front-end. The mass-market receiver is exceptionally robust against the out-of-band chirp, indicating NB operation. The matched spectrum chirp (Figure 14e) has the worst performance among the chirp signals for all of the receivers. It shows the efficiency of this type of chirp: it maximally overlaps with the GNSS signals forcing interference mitigation to remove the GNSS signal along with it.

Figure 15 shows the $C/N_{0}$ for the frequency hopper and pulsed signals. The 12 bit HDDM has the best performance against the frequency hopping interferences (Figure 15a,b). Once again, similar performance to the chirp signals can be asserted. All HDDM implementations outperform the high-end receiver in the pulsed scenarios (Figure 15c,d). It shows that the HDDM is also suitable against pulsed signals, demonstrating the versatility of the mitigation method. For example, at an JSR of 60 dB, the 12 bit HDDM still has a $C/N_{0}$ of 42 dBHz. For the pulsed signals, the difference of the word length for the HDDM implementations seems to have an impact of only a couple of decibels. It also shows that the high-end receiver most likely uses Fourier techniques like FDAF as interference mitigation.

Figure 16 shows the $C/N_{0}$ for the CW signals. In Figure 16a,b, only the HDDM is evaluated, and the NFs are switched off. Here it can be seen that the high-end receiver outperforms all others. It demonstrates the frequency selectivity limitation of the HDDM method. In Figure 16c,d, the HDDM in conjunction with NF is done. The results are improved, showing the synergy of these mitigation algorithms. However, the NF are switched on too early resulting in poor performance at low JSR. This loss indicates that further optimizations of the NF are needed.

## 6. Discussion

In the FMCW (Figure 14) and frequency hopping tests (Figure 15) the 12 bit HDDM shows stable tracking for at least 10 dB higher JSR compared to the 4 and 8 bit HDDM implementations. This difference is a significant improvement and emphasizes the importance of adequate dynamic range. Once the received signal is in saturation, due to the limited dynamic range, the harmonic distortion caused by clipping restricts any interference mitigation. The 4 and 8 bit HDDM go into saturation much sooner; hence, the RFFE and ADC hinder the performance. For the same interference signals, the difference between the 4 and 8 bit HDDM implementations are less than 3 dB. However, in the 4 bit HDDM, the interference-free (i.e., low JSR) tracking is up to 5 dB poorer, compared to the other two implementations. This loss further indicates some limitations caused by limited word-length. These effects are especially visible in the out-of-band chirp (Figure 14d), as the GNSS signals are spectrally isolated from the interference and should theoretically have no impact. It emphasizes the importance of the RFFE design and the selection of the receiver’s analog bandwidth.

All three HDDM implementations exhibit similar performance against pulsed interferences (Figure 15). The interference, and the clipping caused by the interference, are only temporarily present. Hence the word-length limitations are not as severe. Further, all three implementations could suppress the pulsed interferences of at least 50 dB JSR. However, at a certain point, the receivers were still affected by the large interference power. It could be that when no pulse is transmitted, some oscillator leakage of the signal generator may still affect the spectrum.

The HDDM has dissatisfactory performance against simple CW interferences (Figure 16). It is caused by the limited frequency selectivity of the method (see Figure 6) and limits the robustness of the HDDM. However, by combining the HDDM with a simple NF circumvents the issue. This synergy illustrates the benefit of combining several mitigation algorithms together for improved performance. Fundamentally, no single algorithm can perform optimally against every type of interference. Therefore, such synergies should be identified and used for resilient interference mitigation. The HDDM can efficiently remove FMCW signals. Therefore, there is no need to use an ANF, and a simple slow-adaption NF would suffice. Moreover, timing requirements on WB receivers and adaption instabilities of the ANF would further unnecessarily complicate the implementation.

The high-end receiver showed good performance for all but the pulsed interferences. In most cases, the 12 bit HDDM had similar or improved results, indicating that the method is comparable to the current state of the art. The joint NF and 12 bit HDDM showed superior results in most cases, suggesting superior mitigation capability. However, as soon as the word-length is reduced (i.e., 4 or 8 bit), the HDDM shows significant degradation. This degradation challenges the adaption capability of the HDDM to low or medium-end receivers. The 4 bit HDDM had 3 to 10 dB improvement compared to the mass-market receiver, implying that there is still a benefit to include interference mitigation on low-end devices. The mass-market receiver typically had a lower performance to the no-mitigation receiver. This difference indicates that it most likely uses a low ADC word-length. However, the mass-market receiver had a good performance against the out-of-band chirp (Figure 14d), indicating that it is an NB receiver with good analog signal suppression.

In most cases, the high-end receiver stayed in tracking throughout the entire interference test, with the lowest $C/N_{0}$ of 24 dBHz. Similarly, the mass-market receiver often stayed in lock below 20 dBHz. The experimental receivers used by the HDDM seldom stayed in tracking below 30 dBHz. These differences in performance are attributed to the tracking algorithms used and not interference mitigation. It emphasizes the importance of the receiver architecture and all processing stages of the receiver, including interference mitigation, signal acquisition, tracking, and PVT calculation. Furthermore, this difference indicates that the high-end receiver uses integration-lengths (coherent or incoherent) beyond the 4 ms of the PRN sequence for Galileo E1BC. An example is to exploit the secondary code of E1C, to allow up to 100 ms coherent integration. The generated scenario was stationary. In a dynamic scenario, further differences between the various receivers are also expected, as integration lengths are often limited by the dynamics.

## 7. Conclusions

A hardware implementation of the HDDM was presented in this paper. A comparison of three designs, with varying word-lengths, resource requirements, and performance, shows the scalability of the method. The implementations are efficient and require sufficiently low resources to be realized on a single, medium-sized FPGA device.

The implementations are verified using different interference signals. The performance results of the HDDM indicate a limitation to the dynamic range of the receiver: designs with large word-lengths showed superior performance to the current state of the art. On the contrary, designs with low word-lengths showed inferior results compared to a high-end receiver, yet still superior to a mass-market receiver. The HDDM algorithm showed good mitigation capabilities against FMCW, frequency hopping, and pulsed signals. Therefore, it is a versatile method with a high degree of resilience. A significant limitation is the algorithm poor performance against simple CW interferences. Combining the HDDM with simple NFs has shown to adequately address this limitation, proving a good synergy of the two methods. The efficient hardware implementation, together with the performance, indicates good potential for the adaption of the algorithm to current medium and high-end receivers. This approach is a robust method and—depending on the interference type—is comparable or superior to current state-of-the-art high-end receivers. For the 12 bit implementation, with JSRs of 50 to 65 dB the signals still remained in tracking.

Currently, an HDDM using a 32-point FFT is realized. Therefore, it is suggested to investigate the performance and hardware capabilities using an FFT of size 64 or larger. Furthermore, there is still room to optimize the algorithm or to improve performance, for example, to implement non-linear functions instead of PB. Even though many interferences have been evaluated in this paper, there are still other waveforms that can be investigated. These include specific waveforms for communication (e.g., the amateur radio band overlaps in the E6 GNSS band) and ranging systems (e.g., DME in the E5/L5 GNSS band), or other generic waveforms such as band-limited noise signals. Lastly, only the tracking $C/N_{0}$ is evaluated in this study; hence, it is suggested to also look at the PVT performance in future research.

## Figures and Tables

**Table 1:** Device properties and associated resource allocations.

Receiver Number		# 1	# 2	# 3
Device Description		14 bit	8 bit	4 bit
Xilinx SoC device		Zynq7045	ZynqUS+ 9EG	ZynqUS+ 9EG
Input word-length [bits]		14	8	4
PB word-length [bits]		12	12	12
Output word-length [bits]		8	4	4
Baseband sampling frequency $f_{s}$ [MHz]		108	62.5	62.5
Max. PL dynamic power [W]		4.3	2.5	2
Max. PL static power [W]		0.3	0.7	0.7
Module size		N/A	80 × 60 × 15 mm	80 × 60 × 15 mm
Component	Resource	# 1	# 2	# 3
Signal Conditioning	Slice LUTs	~24,000	~16,600	~23,000
	DSP	317	219	153
HDDM	Slice LUTs	~17,000	~14,000	~14,000
	DSP	157	159	129
Mixer	Slice LUTs	19	~700	~700
	DSP	0	0	0
LPF	Slice LUTs	~6000	~2000	~6000
	DSP	152	36	0
NF	Slice LUTs	239	196	210
	DSP	8	8	8

**Table 2:** COTS receivers used in the experimental setup.

	Mass-Market Receiver	High-End Receiver
Price	54	895 (eval. kit)
Power consumption (typ.)	150 mW	600 mW
Module size	17.0 × 22.4 × 3.5 mm	31 × 31 × 4 mm
Interference mitigation capabilities	Unknown	Wideband and CW interferer mitigation

## Abbreviations

The following abbreviations are used in this manuscript:

ADC	analog-to-digital converter
AltBOC	alternative binary offset carrier
ANF	adaptive notch filtering
ARM	Acorn RISC Machine
ASIC	application-specific integrated circuit
BOC	binary offset carrier
BPSK	binary phase-shift keying
$C/N_{0}$	carrier-to-noise density ratio
CORDIC	coordinate rotation digital computer
COTS	commercial-off-the-shelf
CW	continuous-wave
DFT	discrete Fourier transform
DME	distance measurement equipment
DSP	digital signal processor
Dual-FDAF	dual-channel frequency-domain adaptive filtering
FBPB	filter-bank pulse blanking
FDAF	frequency-domain adaptive filtering
FFT	fast Fourier transform
FLL	frequency-locked loop
FM	frequency-modulation
FMCW	frequency-modulated continuous-wave
FPGA	field-programmable gate array
GNSS	global navigation satellite system
HDDM	high-rate DFT-based data manipulator
HW	hardware
IDFT	inverse discrete Fourier transform
IF	intermediate-frequency
IFFT	inverse fast Fourier transform
IIR	infinite impulse response
IP	intellectual property
ISR	interference-to-signal ratio
JSR	jamming-to-signal ratio
KLT	Karhunen-Loève transform
LMS	least mean squares
LO	local oscillator
LPF	low pass filter
LUT	look-up table
ML	machine learning
NB	narrow-band
NF	notch filter
PB	pulse blanking
PC	personal computer
PL	programming logic
PNT	positioning, navigation, and timing
PPD	privacy protection device
PRN	pseudo-random noise
PS	processing system
PSD	power spectral density
PVT	position, velocity, and time
RF	radio-frequency
RFCS	radio-frequency constellation simulator
RFFE	radio-frequency front-end
RTL	register-transfer level
SDR	software-defined radio
SoC	system-on-chip
SSC	spectral separation coefficient
STFT	short-time Fourier transform
SW	software
SWAP-C	size, weight, power, and cost
VHDL	very-high-speed integrated circuit hardware description language
WANF	wavelet-based adaptive notch filter
WB	wide-band
